# ﻿Systematic notes on three new *Luthela* (Mesothelae, Heptathelidae) spiders from China, with their descriptions

**DOI:** 10.3897/zookeys.1159.90120

**Published:** 2023-05-02

**Authors:** Mian Wei, Shuqiao Wang, Yucheng Lin

**Affiliations:** 1 Key Laboratory of Bio-resources and Eco-environment (Ministry of Education), College of Life Sciences, Sichuan University, Chengdu, Sichuan 610064, China Sichuan University Chengdu China; 2 The Sichuan Key Laboratory for Conservation Biology of Endangered Wildlife, Sichuan University, Chengdu, Sichuan 610064, China Sichuan University Chengdu China

**Keywords:** Burrowing spider, COI, heptathelids, molecular analysis, new species, taxonomy

## Abstract

Three new segmented trapdoor spider species belonging to the family Heptathelidae Kishida, 1923, i.e., *Luthelaasuka***sp. nov.** (♂♀, Sichuan), *L.beijing***sp. nov.** (♂♀, Beijing), and *L.kagami***sp. nov.** (♂♀, Sichuan), are described from China. Their phylogenetic position and relationships within Heptathelidae are tested and assessed using a combination available COI data downloaded from GenBank with new DNA sequences obtained in this study. The results show that the new species form a clade with eight known and one undescribed species of *Luthela*. High-definition illustrations of the male palps and female genitalia, diagnoses, and DNA barcodes are provided for these three new species, and their distributions are mapped.

## ﻿Introduction

Mesotheles, commonly known as primitively segmented spiders, are characterized by having a series of plates on the abdomen and the spinnerets situated in the middle of ventral abdomen. The suborder Mesothelae previously included only one extant family Liphistiidae Thorell, 1869 (*s.l.*), which has now been split into two closely related families, Heptathelidae Kishida, 1923 and Liphistiidae Thorell, 1869 (*s.s.*) (Petrunkevitch 1939).

The family Heptathelidae currently consists of 107 extant species in seven genera, whose range is limited to the Far East, such as in Japan, the Ryukyu Islands, China, and Vietnam (Xu et al. 2021; WSC 2023). This family was originally described as a tribe (Heptatheleae) of Liphistiidae (*s.l.*) by Kishida (1923), and subsequently was elevated to the level of a family by Petrunkevitch (1939) and confirmed by Haupt (1983). Raven (1985) synonymized Heptathelidae with Liphistiidae (*s.l.*). Recent molecular phylogenetic studies (Xu et al. 2015a, 2015b, 2021) have confirmed the monophyly of Liphistiidae (*s.l.*) as well as that of its two subfamilies, Heptathelinae and Liphistiinae. Li (2022) restored the subfamily Heptathelinae to the family level and circumscribed Liphistiidae (*s.s.*) to include only all extant species of *Liphistius* Schiødte, 1849. Based on extensive comparisons of the estimated divergence time in extant spider families and known fossils, Breitling (2022) suggested that it would make more sense to reunite both families into Liphistiidae (*s.l.*). WSC (2023) took note of Breitling’s viewpoint, but at present rejected his proposal on the grounds that the age of splitting is not sufficient reason to reunite the families.

*Luthela* Xu & Li, 2022, an endemic genus of northern China, was newly erected and delimited on the basis of morphological characters and molecular data, and it was transferred from Liphistiidae to Heptathelidae (Li 2022; Xu et al. 2022). At present, *Luthela* includes eight known extant species, which are distributed almost exclusively north of the Yangtze River to the Yellow river basin in China, but no species have been recorded in Beijing and Sichuan.

The aims of this paper are 1) to describe and illustrate the three new species; 2) to provide the COI sequences of them for verifying their sex pairing; 3) to test their phylogenetic position and relationships within heptathelids; and 4) to map the geographic distributions of these extant *Luthela* species. This paper expands the knowledge of species diversity of Chinese Heptathelidae.

## ﻿Materials and methods

### ﻿Specimens sampling

Specimens studied here were collected from Beijing City and Sichuan provinces, China, on 8 October 2019, 15 June 2022, 16 October 2022, and 30 January to 1 February 2023. All specimens were captured by hand and stored in 95% ethanol at −20 °C.

### ﻿Molecular data

To test the taxonomic position of the three *Luthela* species, five individuals were selected from the examined materials for molecular data collection. The first and second legs on the right were used to extract genomic DNA and sequence the gene fragments COI. The rest of the bodies were kept as vouchers. All molecular data were obtained from specimens collected at the type localities of the species, although not from the type specimens themselves. Whole genomic DNA was extracted from tissue samples with the Universal Genomic DNA Kit (CWBIO, Beijing, China) following the manufacturer’s protocol for animal tissue. The COI gene fragments were amplified in 50 µL reactions. Primer pairs and PCR protocols are given in Table 1. Raw sequences were edited and assembled using Mesquite v. 3.02 (Maddison and Maddison 2011). New sequences were deposited in GenBank (Table 2). All molecular vouchers and examined materials are stored in the Natural History Museum of Sichuan University in Chengdu, China (**NHMSU**).

**Table 1.** Loci, primer pairs, and PCR protocols used here.

Loucus	Annealing temperature/time	Direction	Primer	Sequence 5ʹ → 3ʹ	Reference
COI	49 °C/15 s	F	LCO1409	GGTCAACAAATCATAAAGATATTGG	Folmer et al. 1994
		R	HCO2198	TAAACTTCAGGGTGACCAAAAAATCA	

To place these new species in a proper taxonomic position within Heptathelidae and verify their sexual pairing, we used these sequences and a selection from previously sequenced taxa to assemble a phylogeny of heptathelid spiders: *Ganthela* Xu & Kuntner, 2015, *Heptathela* Kishida, 1923, *Luthela*, *Qiongthela* Xu & Kuntner, 2015, *Ryuthela* Haupt, 1983, *Songthela* Ono, 2000, and *Vinathela* Ono, 2000. In addition, a *Liphistius* species was used as the outgroup (Table 2). Sequences were aligned with MAFFT v. 7.505 (Katoh and Standley 2013) using ‘-auto’ strategy and normal alignment mode. Best partitioning scheme and evolutionary models for three predefined partitions were selected using PartitionFinder2 v. 2.1.1 (Lanfear et al. 2017), with all algorithms and Akaike information criterion (AIC). SYM+I+G, HKY+I+G, and GTR+G were selected for the first, second, and third codon positions of COI, respectively.

**Table 2.** List of segmented spider taxa and their COI data used for phylogenetic analysis of heptathelids (including five new DNA sequence data obtained here).

Species	Identifier	COI	Species	Identifier	COI
*Liphistiusdesultor*	LS054	KR028518	*Vinathelacucphuongensis*	XUX-2013-008	KT767580
*Ganthelacipingensis*	XUX-2013-516	KP875509	*Vinathelanenglianggu*	DQ-2018-036	MN400648
*Ganthelajianensis*	XUX-2013-534	KP875503	*Luthelabadong*	XUX-2012-140	KP229863
*Ganthelaqingyuanensis*	XUX-2012-288	KP875525	*Lutheladengfeng*	XUX-2012-031	MH172686
*Ganthelavenus*	XUX-2013-160	KP875483	*Luthelahandan*	XUX-2011-214	KP229810
*Ganthelawangjiangensis*	XUX-2012-278	KP875508	*Luthelaluotianensis*	XUX-2012-079	KP229881
*Ganthelaxianyouensis*	XUX-2013-153	KP875526	*Luthelaschensiensis*	XUX-2011-273	MH172701
*Heptathelakimurai*	XUX-2013-356	MN274707	*Luthela* sp.	XUX-2016-110	MH172699
*Heptathelatokashiki*	XUX-2014-051	MN274727	*Luthelataian*	XUX-2014-143A	MH172722
*Qiongthelabaishensis*	XUX-2012-087	KP229805	*Luthelayiyuan*	XUX-2012-051	MH172727
*Qiongthelaqiongzhong*	XUX-2017-156	MN911987	*Luthelayuncheng*	XUX-2011-235	MH172738
*Ryuthelanishihirai*	OKR19	AB778138	*Luthelaasuka* sp. nov.	WM-2019-A002	OQ661856
*Ryuthelaunten*	XUX-2012-531	MF078619	*Luthelaasuka* sp. nov.	WM-2023-A003	OQ661857
*Songthelabristowei*	XUX-2012-256	KP229808	*Luthelabeijing* sp. nov.	WM-2022-B001	OQ661858
*Songthelaciliensis*	XUX-2012-177	KP229918	*Luthelakagami* sp. nov.	WM-2023-K001	OQ661859
*Songthelahangzhouensis*	XUX-2013-171	KT767579	*Luthelakagami* sp. nov.	WM-2023-K002	OQ661860

Bayesian phylogenetic inference (BI) was performed using MrBayes v. 3.2.7 (Ronquist et al. 2012) through Phylosuite v. 1.2.3 (Zhang et al. 2020) using four Markov Chain Monte Carlo (MCMCs) chains with default heating parameters for 50,000,000 generations or until the average standard deviation of split frequencies was <0.01. Markov chains were sampled every 5000 generations, and the first 25% of sampled trees were burn-in. The website iTOL v. 6.7 (Letunic and Bork 2021) was used to analyse the performance of our BI analyses. Maximum-likelihood (ML) phylogenies were also inferred using IQ-TREE v. 2.0 (Nguyen et al. 2015) through Phylosuite v. 1.2.3 (Zhang et al. 2020) under Edge-linked partition model for 1000 ultrafast (Minh et al. 2013) bootstraps, as well as the Shimodaira–Hasegawa-like approximate likelihood-ratio test (Guindon et al. 2010).

### ﻿Morphological data

Specimens were examined and measured with a Leica M205 C stereomicroscope. All male palps and female genitalia were dissected from the bodies before being examined and photographed. To reveal the internal structure, female genitalia were boiled for 5 min in KOH solution (1 mol/L) at 45 °C, and then a dissection needle was used to remove the remaining soft tissue before being photographed. Photographs of male palps and female genitalia were taken with a Canon EOS 60D wide zoom digital camera (8.5 megapixels) mounted on an Olympus BX 43 compound microscope. The images were montaged using Helicon Focus v. 7.0.2 image stacking software (Khmelik et al. 2006). All measurements are given in millimeters. Eye diameters were measured as the maximum diameter in either dorsal or frontal views. Leg measurements are given in the following sequence: total length (femur, patella + tibia, metatarsus, tarsus). Body length was measured only from the anterior edge of prosoma to the posterior edge of opisthisoma, excluding the chelicerae.

Abbreviations used in the text or figures as follows:

**ALE** anterior lateral eyes;

**AME** anterior median eyes;

**ASC** apical spine of conductor;

**BSC** basal spine of conductor;

**Co** conductor;

**CT** contrategulum;

**DT** dorsal extension of TA;

**E** embolus;

**EO** embolus opening;

**MA** marginal apophysis of tegulum;

**MH** middle haematodocha;

**PC** paracymbium;

**PLE** posterior lateral eyes;

**PME** posterior median eyes;

**RC** receptacular cluster;

**ST** subtegulum;

**T** tegulum

**TA** terminal apophysis of tegulum.

## ﻿Results

### ﻿Phylogenetic analysis

**Figure 1.**
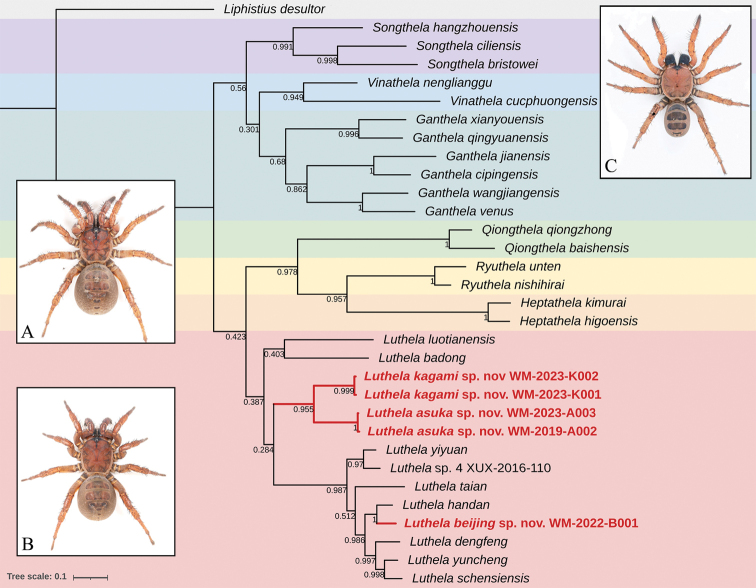
Tree topology obtained by Bayesian analysis in MrBayes v. 3.2.7. Numerical values at nodes indicate posterior probabilities. Note: 29 species representing the family Heptathelidae were clustered into a monophyletic group; the high support of three new species (red font) in the genus *Luthela* (pink box), and the low support of monophyly of 12 *Luthela* species. *Liphistiusdesultor* (light grey box) of Liphistiidae was selected as outgroup for this phylogenetic analysis. Habitus images: **A***Luthelaasuka* sp. nov. **B***Luthelakagami* sp. nov. **C***Luthelabeijing* sp. nov. Photographs by Yejie Lin.

The BI analysis of the dataset of COI genes recovered a single parsimonious tree topology. This tree shows heptathelids are monophyletic but with low support. All 29 heptathelid species included are divided into two major clades, and the seven genera they represent formed the following phylogenetic relationships: (*Songthela* + (*Vinathela* + (*Ganthela*)) + (*Luthela* + (*Qiongthela* + (*Ryuthela* + *Heptathela*)))). These seven genera are also monophyletic, with high support in clades of *Songthela*, *Vinathela*, *Qiongthela*, *Ryuthela*, and *Heptathela*, but low support in the *Ganthela* and *Luthela* clades. Three new species (Fig. 1, indicated by red font) are nested within *Luthela*, which is a clade composed of 12 *Luthela* species (Fig. 1, indicated by a pink box). The sex pairing of all three new species were confirmed to be correct and highly supported as separate clades and belong to the genus *Luthela*. The sister group relationship of *Luthelaasuka* sp. nov. and *Luthelakagami* sp. nov. has high support. The same relationship occurs between *Luthelabeijing* sp. nov. and *Luthelahandan*Xu et al., 2022. These results support our taxonomic decision to recognise them as new species and confirm their higher affinities.

**Figure 2.**
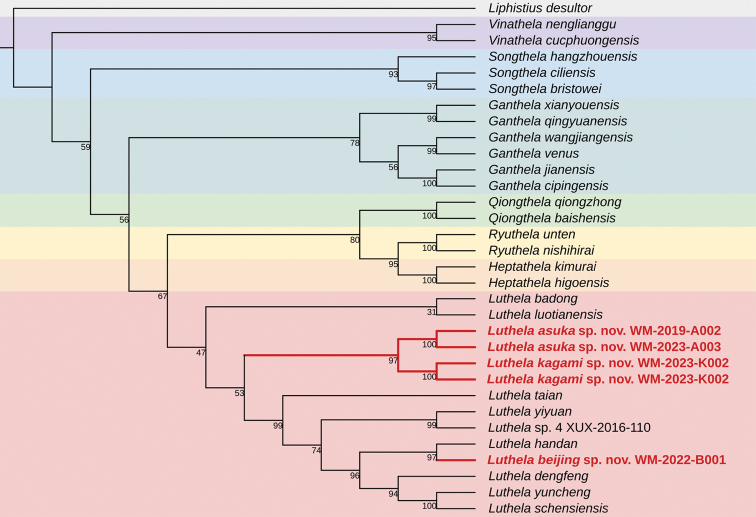
Tree topology obtained by maximum likelihood in IQ-TREE v. 2.0. Numbers at nodes are bootstrap values; other conventions as in Fig. 1. The clade of the three new *Luthela* species (red font) is nested within *Luthela* (pink box). Further clades are other genera of Heptathelidae (*Heptathela*, *Ryuthela*, *Qiongthela*, *Ganthela*, *Songthela*, and *Vinathela* are from the bottom up).

The result of ML is consistent with that of the BI on some major clades, but there are some differences (Fig. 2). In the ML tree, all 29 heptathelid species also clustered into a monophyletic group. Different from the topology structure of BI tree, the phylogenetic relationships of the seven genera they represent are as follows: (*Vinathela* + (*Songthela* + (*Ganthela* + (*Luthela* + (*Qiongthela* + (*Ryuthela* + *Heptathela*)))))). Also, as in the BI tree, the clades of *Vinathela*, *Songthela*, *Qiongthela*, *Ryuthela*, and *Heptathela* have high support, but the clades of *Ganthela* and *Luthela* have low support. As a sister group, the clade of *Luthela* is delimited to include eight known, three new, and one still undescribed species. Both BI and ML analyses show that the three new species form a clade which is the sister group to remaining *Luthela* species. The available molecular evidence supports the taxonomic placement of the three new *Luthela* species.

### ﻿Taxonomy

**Family Heptathelidae Kishida, 1923**

**Genus *Luthela* Xu & Li, 2022**

#### ﻿ Luthela

Taxon classificationAnimaliaAraneaeHeptathelidae

Xu & Li, 2022: 134.

F379106C-26D4-52CF-A87E-27327931B90B

##### Type species.

*Luthelayiyuan* Xu, Yu, Liu & Li, 2022 by original designation, from Yiyuan Co., Shandong Province, China.

##### Diagnosis.

**Figure 3.**
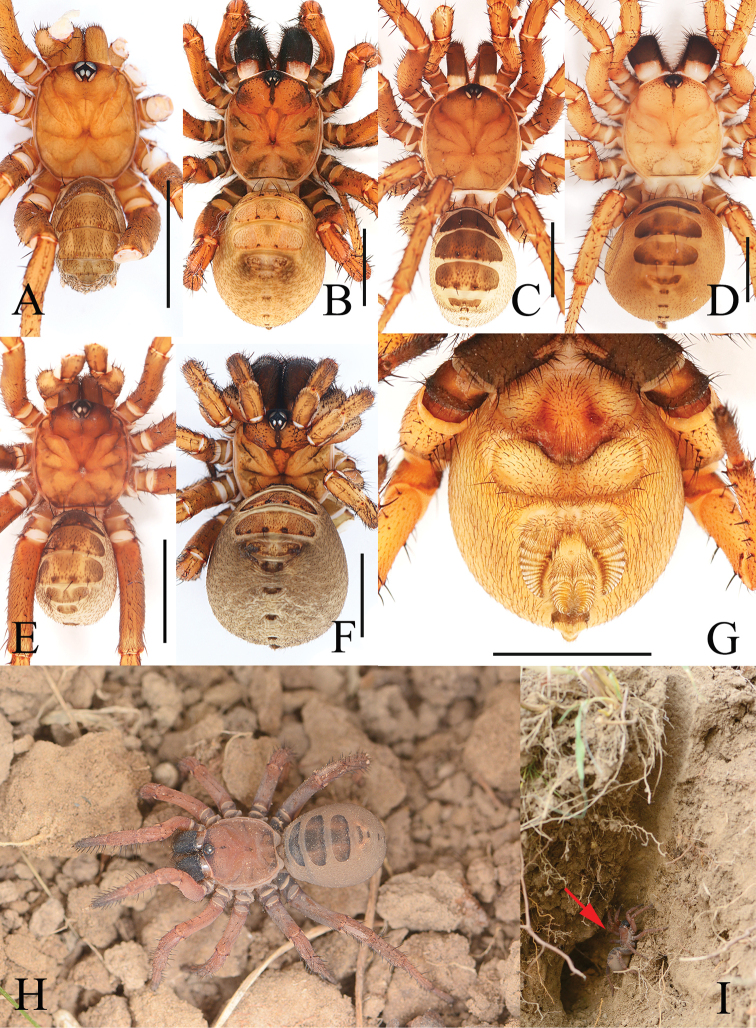
New species of *Luthela***A, B, G***L.asuka* sp. nov. from Longquanyi District, Chengdu **C, D, H, I***L.beijing* sp. nov. from Zizhuyuan Park, Beijing **E, F***L.kagami* sp. nov. from Guihua Township, Pengzhou City **A, C, E** male habitus, dorsal view **B, D, F** female habitus, dorsal view **G** female haibitus, ventral view **H** living female, dorsal view **I** burrow, vertical section, with red arrow pointing to the spider. Photographs by Chao Wu (**H, I**). Scale bars: 5.00 mm.

**Figure 4.**
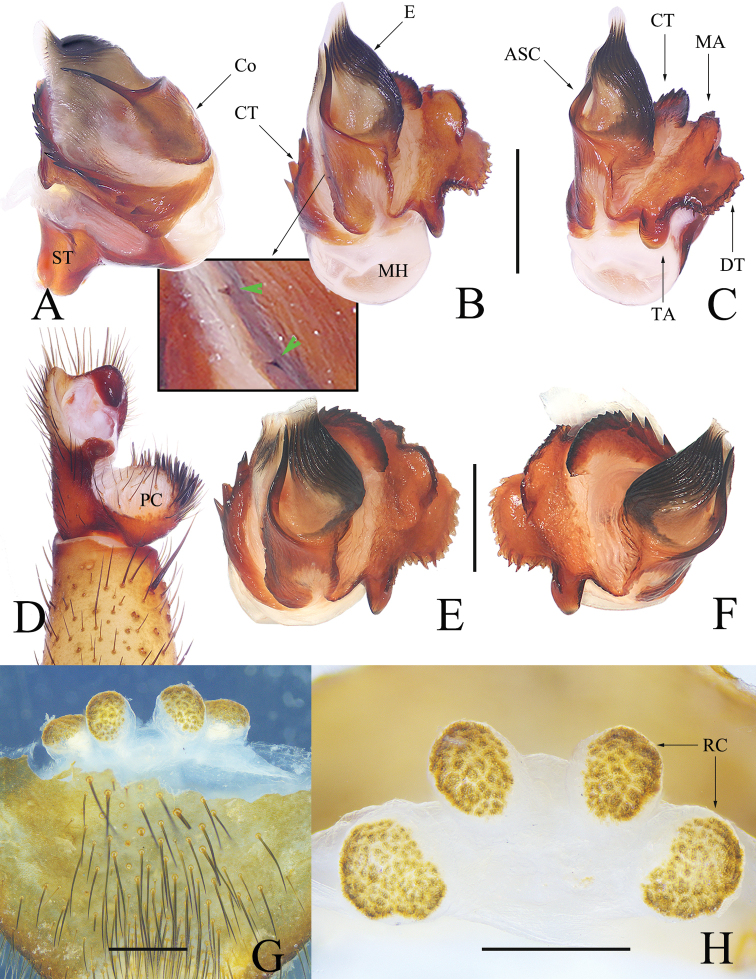
*Luthelaasuka* sp. nov. **A** male left palp bulb, prolateral view **B** male left palp bulb, ventral view **C** male left palp bulb, retrolateral view **D** left cymbium, ventral view **E** left palpal bulb, apical view **F** right palpal bulb, apical view **G** vulva, ventral view **H** vulva, dorsal view. Green arrows indicate small teeth on conductor. Scale bars: 0.50 mm.

**Figure 5.**
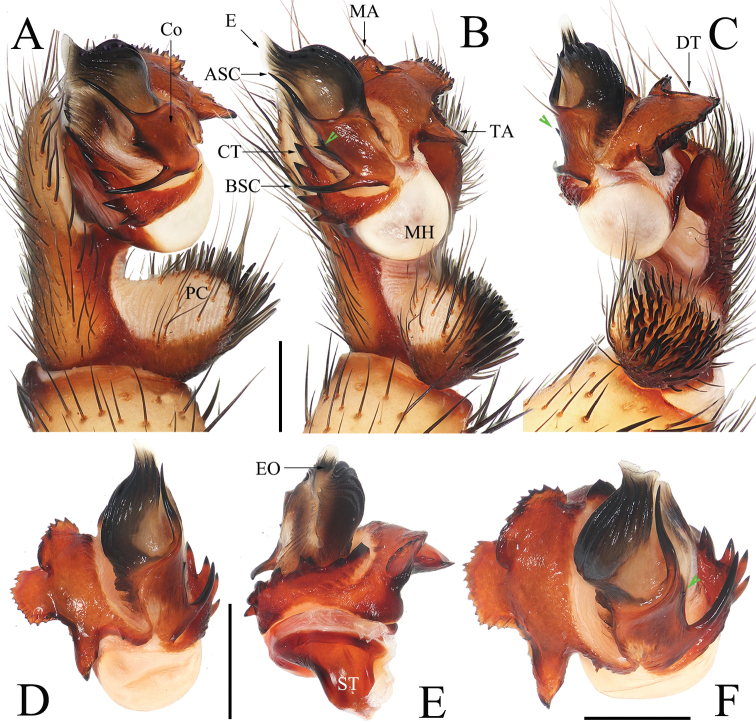
*Luthelabeijing* sp. nov., male holotype **A** left palp, prolateral view **B** left palp, ventral view **C** left palp, retrolateral view **D** right palpal bulb, ventral view **E** right palpal bulb, dorsal view **F** right palpal bulb, apical view. Green arrows in **B, C**, and **F** indicate small teeth on conductor. Scale bars: 0.50 mm.

**Figure 6.**
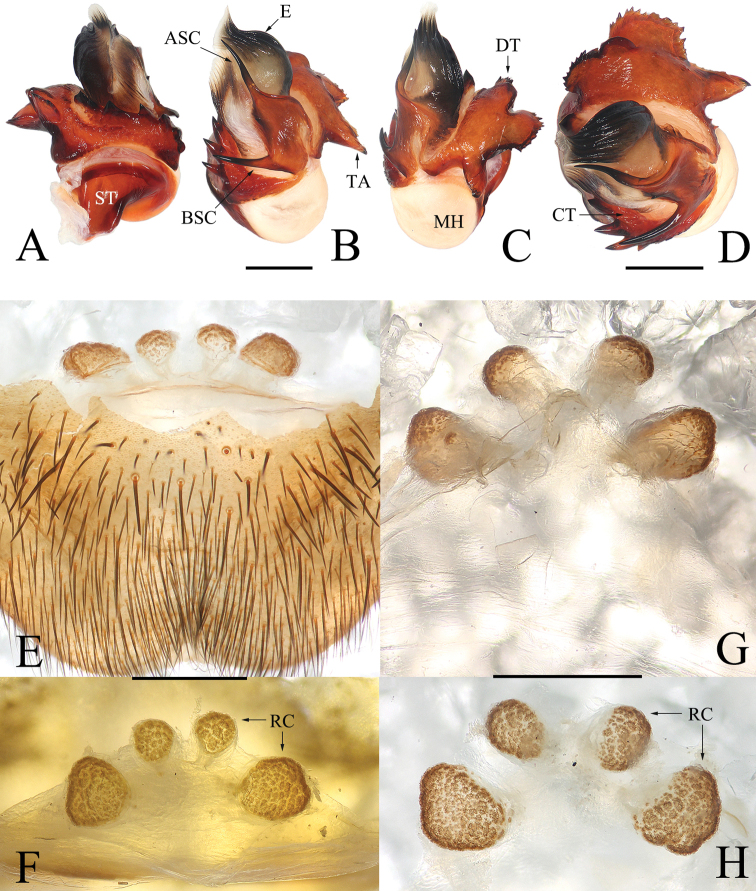
*Luthelabeijing* sp. nov. **A–D** left palpal bulb **E–H** female genitalia **A, F, H** dorsal view **B, E, G** ventral view **C** retrolateral view **D** apical view. Scale bars: 0.50 mm.

Males of *Luthela* differ from those of other heptathelid genera except *Songthela*, by the smooth conductor with one or two long spines (see ASC and BSC in Figs 3C, 4B, 5B, 6B), and they can be distinguished from the males of *Songthela* in having regular larger teeth on the contrategular margin (see CT in Figs 3B, 3C, 4B, 5D, 6B, 6C). Females of *Luthela* can be recognized from those of other genera by the middle pair of the receptacular clusters being situated at the anterior margin of the bursa copulatrix and the lateral ones at the dorsolateral position of the bursa copulatrix (Fig. 3H, 5F, 5H, 6H).

##### Composition.

*Luthelaasuka* Wei & Lin, sp. nov. (♂♀, Sichuan), *Luthelabadong*Xu et al., 2022 (♂♀, Hubei), *L.beijing* Wei & Lin, sp. nov. (♂♀, Beijing), *L.dengfeng*Xu et al., 2022 (♂♀, Henan), *L.handan*Xu et al., 2022 (♂♀, Henan), *Luthelakagami* Wei & Lin, sp. nov. (♂♀, Sichuan), *L.luotianensis* (Yin et al., 2002) (♀, Hubei), *L.schensiensis* (Schenkel, 1953) (♂♀, Shaanxi), *L.taian*Xu et al., 2022 (♂♀, Shandong), *L.yiyuan*Xu et al., 2022 (♂♀, Shandong), and *L.yuncheng*Xu et al., 2022 (♂♀, Shanxi).

##### Distribution.

Northern China, from the Yangtze River to the Yellow river basin.

#### ﻿ Luthela asuka

Taxon classificationAnimaliaAraneaeHeptathelidae

Wei & Lin sp. nov.

A92F27A4-AA61-5477-BDD0-879EAB711980

https://zoobank.org/917ACCF0-7506-496C-8C29-0CF53D0C710D

Figs 3A, B, G , 4

##### Type material.

***Holotype*** ♂, **China**: Sichuan Province, Chengdu City, Longquanyi District, Longquan Mountain Forest Park, near Tiangong Temple, 30.5305°N, 104.2709°E, 636 m elev., 8.X.2019, M. Wei and Y. Shen leg. ***Paratypes*** 1♀, **China**: Sichuan Province, Chengdu, Longquan District, Longquan Mountain Forest Park, near the expressway of Chengdu to Jianyang, 30.5381°N, 104.3015°E, 740 m elev., 16.X.2022, S. Wang leg.; 1♀, **China**: Sichuan Province, Chengdu, Longquan District, Longquan Mountain Forest Park, near the expressway of Chengdu to Jianyang, 30.5381°N, 104.3015°E, 740 m elev., 1.II.2023, S. Wang and M. Wei leg. Deposited in NHMSU.

##### Etymology.

The specific epithet is from “Asuka Langley Soryu”, a fictional character wearing a red combat suit from the animation “Evangelion” (by the Japanese creator Hideaki Anno), refers to the body color; noun (name) in apposition.

##### Diagnosis.

**Figure 7.**
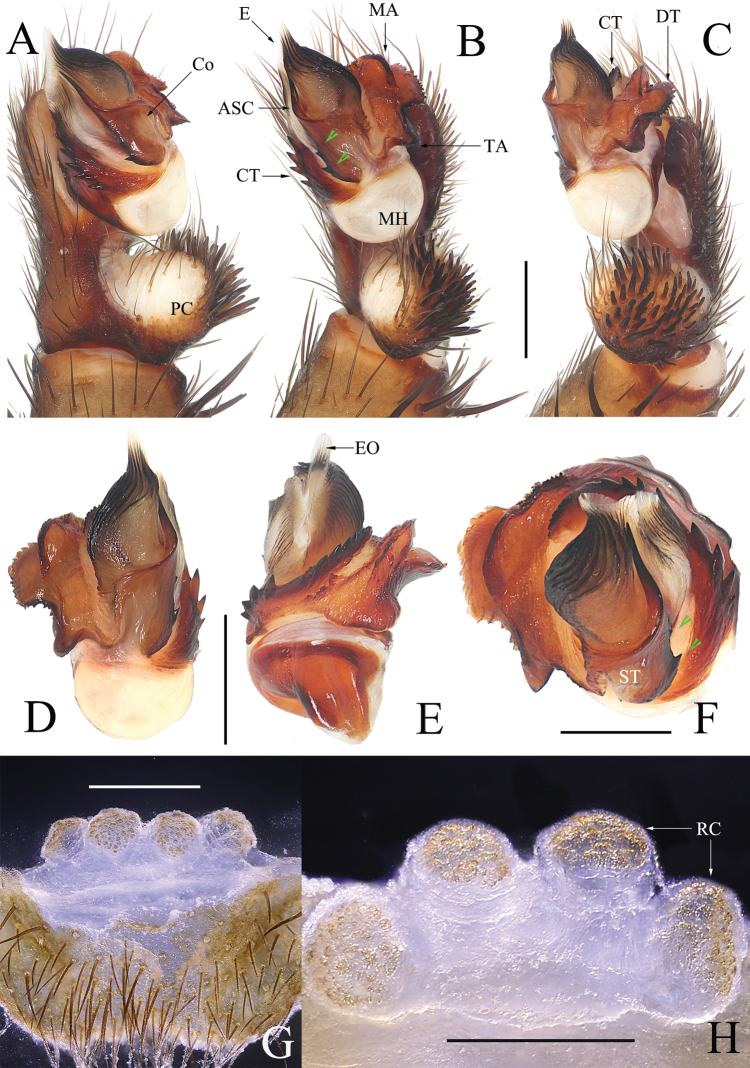
*Luthelakagami* sp. nov. **A** male left palp, prolateral view **B** male left palp, ventral view **C** male left palp, retrolateral view **D** right palpal bulb, ventral view **E** right palpal bulb, dorsal view **F** right palpal bulb, apical view **G** vulva, ventral view **H** vulva, dorsal view. Green arrows in **B** and **F** indicate small teeth on conductor. Scale bars: 0.50 mm.

Males can be distinguished from those of congeners, except *L.kagami* Wei & Lin, sp. nov., in lacking the BSC (Fig. 4A), contrary to other species (cf. Xu et al. 2022: figs 3B, 5E, 6B, 7E, 10B, 12B, 14D), and in having the contrategulum bearning relatively dense, smaller serrated teeth (Fig. 4B, E), rather than sparse and larger teeth in other species (cf. Xu et al. 2022: figs 3A, 5D, 6B, 7D, 10H, 12D, 14H). Males also differ from *L.kagami* sp. nov. in having two nearly invisible lateral teeth on the middle portion of the conductor and the longer TA (Fig. 4A–C, E, F), rather than two relatively larger teeth and a shorter TA in the latter (Fig. 7B–D, F). Females differ from those of congeners in having the paired receptacular clusters situated at the relatively short genital stalks and in the relatively smaller size (Fig. 4G, H), rather than the long genital stalks and the larger size (cf. Xu et al. 2022: figs 4, 5H, I, 6H–M, 8, 9, 11, 13, 14H–M). Females differ from those of *L.kagami* sp. nov. in having the receptacular clusters relatively separated and the lateral pair larger than the middle pair (Fig. 4G, H), rather than closer and nearly equal in size (Fig. 7G, H).

##### Description.

**Male** (holotype) (Fig. 3A). Carapace red; cervical and radial groove distinct. Cephalic region moderately raised. Chelicerae robust; fang furrow with 11 promarginal teeth of variable size. Sternum longer than wide. Abdomen pale yellow, with 5 large dorsal and 2 small posterior tergites, 4 tapering setae near posteromargin of 5 large tergites, and 2 on the rest. Seven spinnerets. Measurements: body 12.06 long. Carapace 5.59 long, 5.09 wide. Abdomen 5.92 long, 4.51 wide. Sternum 2.49 long, 1.91 wide. ALE > PLE > PME > AME. Leg I 18.00 (4.90 + 5.58 + 4.66 + 2.86), leg II 18.08 (4.54 + 5.42 + 5.04 + 3.08), leg III 19.31 (4.20 + 5.41 + 5.87 + 3.83), leg IV 26.76 (6.21 + 7.70 + 8.10 + 4.76).

***Palp*** (Fig. 4A–F): prolateral paracymbium pale, weakly sclerotized, with numerous setae and spines at distal and retrolateral surface. Contrategular margin denticulate, with large teeth on proximal part and smaller but denser teeth on distal part. Marginal apophysis of tegulum serrated, with tapering terminal apophysis of tegulum, margin of dorsal extension of terminal apophysis with teeth varied in size and distance. Conductor smooth, fused to embolic base, with large apical spine and 2 tiny lateral spines on middle portion. Embolus with translucent, flat opening and several ribbed ridges distally.

**Female** (one of paratypes) (Fig. 3B, G). Carapace red, with dark pattern; cervical and radial grooves distinct, with sparse spines. Cephalic region slightly elevated. Chelicerae more robust than male, fang furrow with 12 promarginal teeth of variable size, larger than male. Sternum longer than wide. Abdomen pale, with five large and five small tergites; chaetotaxy on tergites as in male. Seven spinnerets. Measurements: body 16.12 long. Carapace 7.02 long, 6.94 wide. Abdomen 8.93 long, 8.08 wide. Sternum 3.39 long, 1.86 wide. ALE > PLE > PME > AME. Leg I 14.84 (4.80 + 5.50 + 2.57 + 1.97), leg II 14.96 (4.59 + 4.66 + 3.32 + 2.39), leg III 14.70 (4.64 + 4.61 + 2.94 + 2.51), leg IV 22.24 (6.57 + 6.64 + 5.83 + 3.20).

***Female genitalia*** (Fig. 4G, H). Two pairs of receptacular clusters situated on short and thick stalks; lateral pair relatively larger than middle pair. Middle pair of receptacular clusters separated from each other, situated on anteromargin of bursa copulatrix; lateral receptacular clusters situated slightly dorsolaterally.

##### Distribution.

**Figure 8.**
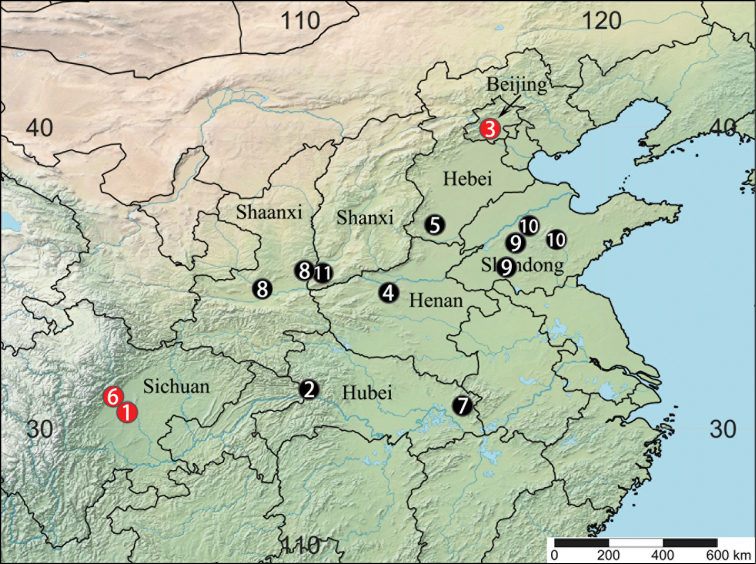
Distribution records of 11 *Luthela* species. 1 = *L.asuka* sp. nov., 2 = *L.badong*, 3 = *L.beijing* sp. nov., 4 = *L.dengfeng*, 5 = *L.handan*, 6 = *L.kagami* sp. nov., 7 = *L.luotianensis*, 8 = *L.schensiensis*, 9 = *L.taian*, 10 = *L.yiyuan*, 11 = *L.yuncheng*.

Known only from the type locality (Fig. 8).

#### ﻿ Luthela beijing

Taxon classificationAnimaliaAraneaeHeptathelidae

Wei & Lin sp. nov.

A781EC6D-C102-5A69-9FAA-FA03D7CF480F

https://zoobank.org/AEB13509-6970-44FE-8CC9-40963F20B516

Figs 3C, D, H, I , 5 , 6

##### Material examined.

***Holotype*** ♂ and ***paratypes*** 1♂ 2♀, **China**: Beijing, Haidian District, near Baishi Bridge, Zizhuyuan Park, 39.9393°N, 116.3110°E, 55 m elev., 15.VI.2022, H. Yang leg. Deposited in NHMSU.

##### Etymology.

The specific epithet derives from the type locality; noun in apposition.

##### Diagnosis.

Males of this new species can be recognized from those of other congeners, except *L.handan*, *L.schensiensis*, *L.yiyuan*, and *L.yuncheng*, by the conductor having 2 spines of nearly equal length and by having a lateral tooth on the middle portion of conductor (Fig. 5B, F), rather than 2 spines in unequal length or lacking a lateral tooth on the conductor (cf. Xu et al. 2022: figs 5B, D, 6A, E, 12A, B, D). Males differ from those of *L.schensiensis* and *L.yuncheng* in having 6 or 7 large teeth on the contrategular (Figs 5F, 6A), rather than 7–10 in *L.schensiensis* and 8 in *L.yuncheng*. (cf. Xu et al. 2022: figs 10G, K, 14D). Males differ from those of *L.yiyuan* by the margin of the contrategular having relatively longer teeth and the distal tooth bifurcated (Figs 5B, 6A, D), rather than shorter teeth on contrategular and the distal tooth with 3 serrations. (cf. Xu et al. 2022: fig. 3G, K). And males differ from those of *L.handan* in having the basal spine of conductor thinner and shorter and the promixal part of the margin of the marginal apophysis with a row of smaller teeth (Figs 5B, F, 6B, D), rather than with a thick, long basal spine on the conductor and the proximal margin of the marginal apophysis with 3 larger teeth. (cf. Xu et al. 2022: fig. 7E, G). Females can be distinguished from those of congeners in having the 2 paired receptacular clusters with longer genital stalks and the lateral pair equal to ca 2× size the middle ones (Fig. 6E–H), rather than shorter genital stalks and the lateral receptacular clusters greater than 3× or less than 2× the middle ones in size (cf. Xu et al. 2022: figs 4, 5H, I, 6H–M, 8, 9, 11, 13, 14).

##### Description.

**Male** (holotype) (Fig. 3C). Carapace black in life; cervical and radial grooves distinct, with sparse spines. Cephalic region moderately raised. Chelicerae robust; fang furrow with 9 promarginal teeth of variable size. Sternum longer than wide. Abdomen pale, with short setae, with 4 large dorsal and 6 small posterior tergites. Four tapering setae near posteromargin of large tergites, 2 on the rest. Seven spinnerets. Measurements: body 14.89 long. Carapace 6.22 long, 5.38 wide. Abdomen 7.82 long, 4.08 wide. Sternum 2.99 long, 1.86 wide. ALE > PLE > PME > AME. Leg I 17.62 (5.13 + 5.26 + 4.46 + 2.77), leg II 17.93 (5.26 + 5.49 + 4.36 + 2.82), leg III 19.34 (4.89 + 5.62 + 5.47 + 3.36), leg IV 25.27 (6.04 + 7.29 + 7.79 + 4.15).

***Palp*** (Figs 5A–F, 6A–D): prolateral paracymbium pale, weakly sclerotized; distal and retrolateral sides with numerous setae and spines. Contrategulum with denticulate margin, with 7 teeth, the fifth bifurcated, and only 4 large teeth visible in dorsal view. Posterior part of marginal apophysis of tegulum serrated, with regular, small denticles; terminal apophysis of tegulum relatively long, apex pointed in distal view, margin of dorsal extension of terminal apophysis with teeth nearly equal in size and distance. Conductor smooth, fused to embolic base, 2 long spines separated at a wide angle, a small tooth located between upper spines and lower spines of conductor. Embolus with translucent, flat opening, and several ribbed ridges distally.

**Female** (one of paratypes) (Fig. 3D, H, I). Carapace red; cervical and radial grooves distinct, with sparse spines. Cephalic region slightly elevated. Chelicerae more robust than male; fang furrow with 10 promarginal teeth of variable size; larger than male. Sternum longer than wide. Abdomen pale, with 4 large and 6 small tergites; chaetotaxy on tergites as in male. Seven spinnerets. Measurements: body 18.12 long. Carapace 7.36 long, 7.29 wide. Abdomen 9.72 long, 7.28 wide. Sternum 3.62 long, 2.49 wide. ALE > PLE > PME > AME. Leg I 15.63 (5.01 + 5.54 + 3.05 + 2.03), leg II 14.80 (4.90 + 4.72 + 3.08 + 2.10), leg III 16.22 (4.99 + 5.29 + 3.70 + 2.24), leg IV 23.23 (6.22 + 7.10 + 6.27 + 3.64).

***Female genitalia*** (Fig. 6E–H). Two pairs of receptacular clusters situated on stalks, middle pair of receptacular clusters separated from each other, on anteromargin of bursa copulatrix, distinctly smaller than lateral pair. Lateral receptacular clusters dorsolateral, stalks thick.

##### Distribution.

Known only from the type locality (Fig. 8).

#### ﻿ Luthela kagami

Taxon classificationAnimaliaAraneaeHeptathelidae

Wei & Lin sp. nov.

7A5E0130-6E2C-5F72-B68D-F50AD80BAB08

https://zoobank.org/20078EAE-3269-428C-8640-54398ACBC00F

Figs 3E, F , 7

##### Type material.

***Holotype*** ♂, **China**: Sichuan Province, Pengzhou, Guihua County, 31.0548°N, 103.8100°E, 664 m elev., 4.X.2021, Y. He leg.; ***paratypes*** 2♀, same data as holotype, 30.I.2023, S. Wang and M. Wei leg. Deposited in NHMSU.

##### Etymology.

The specific epithet is from “Hiiragi Kagami”, a fictional character from the comic “Lucky Star” (written and illustrated by the Japanese cartoonist Yoshimizu Kagami) with haircut similar to “Asuka Langley Soryu” (see Etymology of *Luthelaasuka* sp. nov.); the name refers to the great similarity between these two new species; noun (name) in apposition.

##### Diagnosis.

Males can be distinguished from those of other congeners, except *L.asuka* sp. nov., in lacking BSC (Fig. 7A), in contrast to other species (cf. Xu et al. 2022: figs 3B, 5E, 6B, 7E, 10B, 12B, 14D), and in the contrategulum having relatively dense, smaller serrated teeth (Fig. 7B, F), rather sparse but larger teeth in other species (cf. Xu et al. 2022: figs 3A, 5D, 6B, 7D, 10H, 12D, 14H). Males differ from those of *L.asuka* sp. nov. in having two relatively large teeth on the middle portion of conductor and a shorter TA (Fig. 7B–D, F), rather than with two tiny, nearly invisible teeth and a longer TA (Fig. 4A, B, E). Females differ from congeners, except *L.asuka* sp. nov., in having the paired receptacular clusters with relatively short genital stalks and in their relatively smaller size (Fig. 7G, H), rather than long genital stalks and large size (cf. Xu et al. 2022: figs 4, 5H, I, 6H–M, 8, 9, 11, 13, 14H–M). Females can be distinguished from *L.asuka* sp. nov. in having the receptacular clusters close and nearly equal in size (Fig. 7G, H), rather than separated and with the lateral pair larger than the middle pair (Fig. 4G, H).

##### Description.

**Male** (holotype) (Fig. 3E). Carapace red; cervical and radial grooves distinct. Cephalic region moderately raised. Chelicerae robust; fang furrow with 12 promarginal teeth of variable size. Sternum longer than wide. Abdomen pale yellow, with 5 large dorsal and 2 small posterior tergites, 4 tapering setae near posteromargin of 5 large tergites and 2 on the rest. Seven spinnerets. Measurements: body 11.27 long. Carapace 5.50 long, 4.97 wide. Abdomen 5.77 long, 4.28 wide. Sternum 2.41 long, 1.89 wide. ALE > PLE > PME > AME. Leg I 17.84 (4.87 + 5.53 + 4.62 + 2.82), leg II 17.89 (4.50 + 5.37 + 4.97 + 3.05), leg III 19.19 (4.19 + 5.38 + 5.83 + 3.79), leg IV 26.62 (6.18 + 7.67 + 8.05 + 4.72).

***Palp*** (Fig. 7A–F): prolateral paracymbium pale, weakly sclerotized, with numerous setae and spines at distal and retrolateral sides. Contrategular margin denticulate, with large teeth on proximal part, and smaller but denser teeth distally. Marginal apophysis of tegulum serrated, with relatively short terminal apophysis of tegulum; margin of dorsal extension of terminal apophysis with teeth varied in size and distance. Conductor smooth, fused to embolic base, with large apical spine and 2 small lateral spines on middle portion. Embolus with translucent, flat opening and several ribbed ridges distally.

**Female** (one of paratypes) (Fig. 3F). Carapace red, with dark pattern, cervical and radial grooves distinct, with sparse spines. Cephalic region slightly elevated. Chelicerae more robust than male, fang furrow with 12 promarginal teeth of variable size, larger than male. Sternum longer than wide. Abdomen pale, with 5 large and five 5 tergites; chaetotaxy on tergites as in male. Seven spinnerets. Measurements: body 15.74 long. Carapace 7.00 long, 6.93 wide. Abdomen 8.89 long, 8.21 wide. Sternum 3.24 long, 1.79 wide. ALE > PLE > PME > AME. Leg I 14.70 (4.77 + 5.47 + 2.55 + 1.91), leg II 14.85 (4.57 + 4.63 + 3.30 + 2.35), leg III 14.61 (4.61 + 4.59 + 2.92 + 2.49), leg IV 22.15 (6.54 + 6.63 + 5.82 + 3.16).

***Female genitalia*** (Fig. 7G–H). Two pairs of receptacular clusters on short, thick stalks, close to each other, nearly equal in size. Middle pair of receptacular clusters separated from each other, on anteromargin of bursa copulatrix; lateral receptacular clusters set slightly dorsolaterally.

##### Distribution.

Known only from the type locality (Fig. 8).

## Supplementary Material

XML Treatment for Luthela

XML Treatment for Luthela asuka

XML Treatment for Luthela beijing

XML Treatment for Luthela kagami
